# 3-Chloro-2,4,5-trifluoro­benzoic acid

**DOI:** 10.1107/S1600536812049446

**Published:** 2012-12-08

**Authors:** Jing Quan, Hong-Shun Sun

**Affiliations:** a Department of Applied Chemistry, Nanjing College of Chemical Technology, Geguan Road No. 265 Nanjing, Nanjing 210048, People’s Republic of China; b Chemical Engineering Department, Nanjing College of Chemical Technology, Geguan Road No. 265 Nanjing, Nanjing 210048, People’s Republic of China

## Abstract

The title compound, C_7_H_2_ClF_3_O_2_, was prepared by the chlorination of 3-amino-2,4,5-trifluoro­benzoic acid. The carboxyl group is twisted relative to the benzene ring by 6.8 (1)°. In the crystal, pairs of O—H⋯O hydrogen bonds link mol­ecules into typical centrosymmetric carb­oxy­lic acid dimers. These dimers are arranged into sheets parallel to (-103).

## Related literature

For applications of the title compound in synthesis, see: Sun *et al.* (2011 ▶). For a related structure, see: Zhu (2009 ▶).
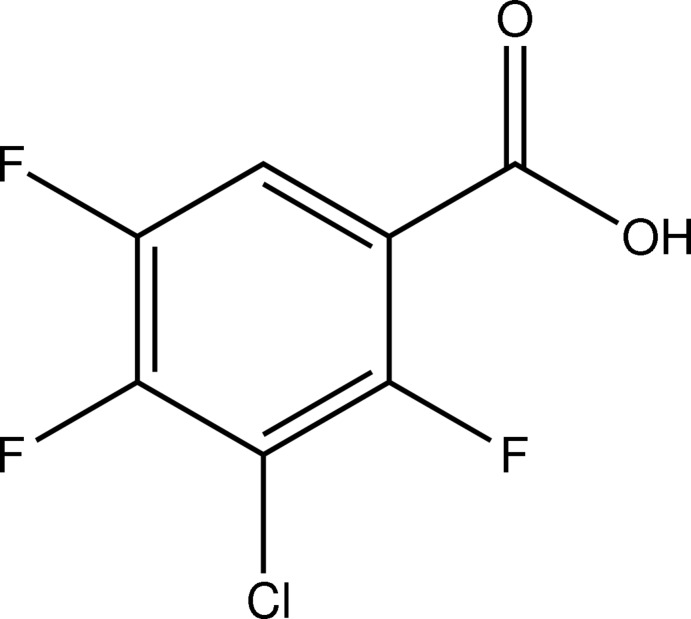


## Experimental

### 

#### Crystal data

C_7_H_2_ClF_3_O_2_
*M* *_r_* = 210.54
Monoclinic, 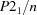

*a* = 4.4760 (9) Å
*b* = 13.654 (3) Å
*c* = 12.400 (3) Å
β = 97.16 (3)°
*V* = 751.9 (3) Å^3^
*Z* = 4
Mo *K*α radiation
μ = 0.52 mm^−1^
*T* = 293 K
0.30 × 0.20 × 0.10 mm

#### Data collection

Enraf–Nonius CAD-4 diffractometer
Absorption correction: ψ scan (North *et al.*, 1968 ▶) *T*_min_ = 0.859, *T*_max_ = 0.950
1578 measured reflections
1394 independent reflections
699 reflections with *I* > 2σ(*I*)
*R*_int_ = 0.092
3 standard reflections every 200 reflections intensity decay: 1%

#### Refinement

*R*[*F* ^2^ > 2σ(*F* ^2^)] = 0.052
*wR*(*F* ^2^) = 0.132
*S* = 1.00
1394 reflections
118 parameters
H-atom parameters constrained
Δρ_max_ = 0.16 e Å^−3^
Δρ_min_ = −0.17 e Å^−3^

### 

Data collection: *CAD-4 EXPRESS* (Enraf–Nonius, 1994 ▶); cell refinement: *CAD-4 EXPRESS*; data reduction: *XCAD4* (Harms & Wocadlo, 1995 ▶); program(s) used to solve structure: *SHELXTL* (Sheldrick, 2008 ▶); program(s) used to refine structure: *SHELXTL*; molecular graphics: *SHELXTL*; software used to prepare material for publication: *SHELXTL*.

## Supplementary Material

Crystal structure: contains datablock(s) I, global. DOI: 10.1107/S1600536812049446/gk2539sup1.cif

Structure factors: contains datablock(s) I. DOI: 10.1107/S1600536812049446/gk2539Isup2.hkl

Supplementary material file. DOI: 10.1107/S1600536812049446/gk2539Isup3.cml

Additional supplementary materials:  crystallographic information; 3D view; checkCIF report

## Figures and Tables

**Table 1:** Hydrogen-bond geometry (Å, °)

*D*—H⋯*A*	*D*—H	H⋯*A*	*D*⋯*A*	*D*—H⋯*A*
O2—H2*A*⋯O1^i^	0.82	1.84	2.658 (4)	178

Symmetry code: (i) 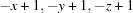.

**Fig. 1.**
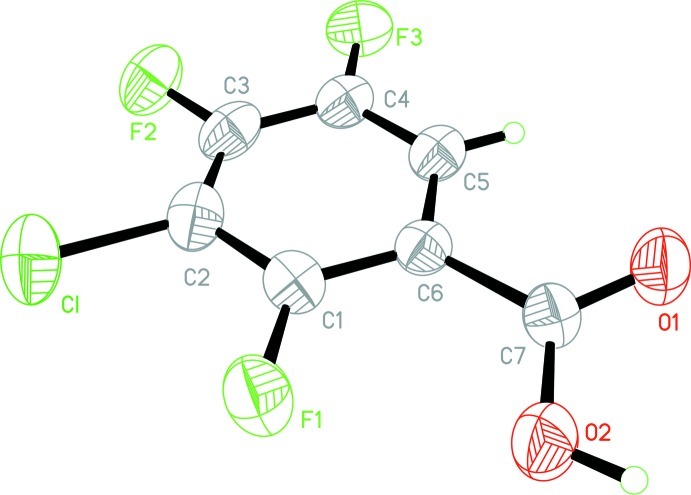
The molecular structure of (I), showing the atom-numbering scheme and displacement ellipsoids at the 30% probability level.

**Fig. 2.**
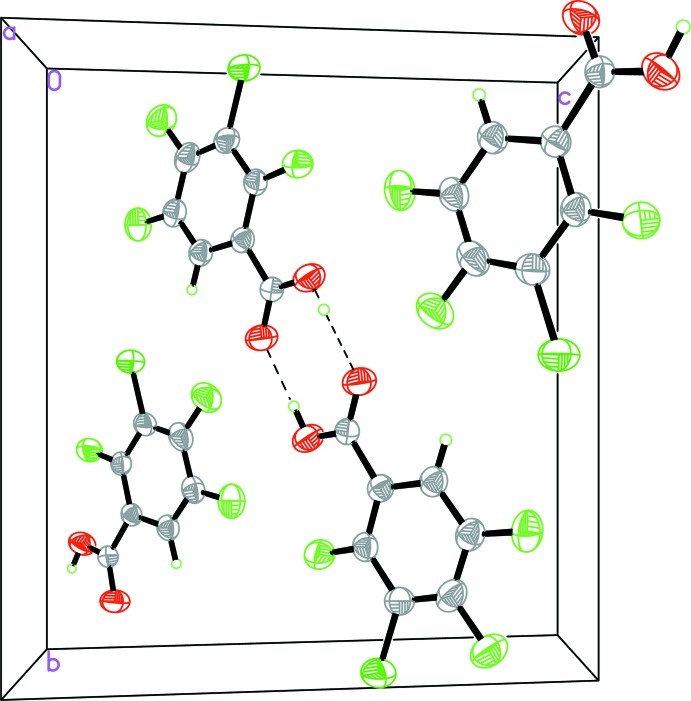
A packing diagram of (I). Intermolecular hydrogen bonds are shown as dashed lines.

## supplementary crystallographic information

### Comment

Ethyl 8-chloro-1-cyclopropyl-6,7-difluoro-4-oxo-1,4-dihydroquinoline- 3-carboxylate is a key intermediate in the preparation of 7-aminosubstituted 8-chloro-1-cyclopropyl-6-fluoro-1,4-dihydro-4-oxo-3-quinolinecarboxylic acids which are useful as antibacterial agents and its crystal structure was recently reported (Sun *et al.*, 2011). In turn 3-chloro-2,4,5-trifluorobenzoic acid, that is not commercially available, is an important material for its preparation. This compound is not easisly synthesized and herein we report its synthesis and the crystal structure.

In the title molecule (Fig. 1) the carboxyl group forms a dihedral angle of 6.8 (1)° with the benzene ring. Intermolecular O—H···O hydrogen bond (Table 1) links the molecules into typical carboxylic acid dimers.

### Experimental

A solid mixture of 0.52 g of 3-amino-2,4,5-trifluorobenzoic acid and 0.33 g of sodium nitrite was added in portions to a solution of 3 g of cupric chloride in 9 ml of water and 0.5 g of a 36% aqueous solution of hydrochloric acid. The resulting mixture was stirred for 1.5 h and then additional water (25 ml) and diethyl ether (20 ml) were added. The layers are separated and the aqueous layer extracted with diethyl ether. The combined organic extracts are extracted with 36% aqueous solution of hydrochloric acid and then concentrated on the rotary evaporator to give 0.45 g of 3-chloro-2,4,5-trifuorobenzoic acid as a light-brown solid. Crystals of the title compound suitable for X-ray analysis were obtained by slow evaporation of a toluene solution.

### Refinement

H atoms were positioned geometrically with O—H = 0.82 Å and C—H = 0.93 Å and constrained to ride on their parent atoms, with *U*_iso_(H) =xUeq(C,*O*), where *x* = 1.5 for OH and *x* = 1.2 for CH H atoms.

### Crystal data

**Table:**

C_7_H_2_ClF_3_O_2_	*F*(000) = 416
*M**_r_* = 210.54	*D*_x_ = 1.860 Mg m^−^^3^
Monoclinic, *P*2_1_/*n*	Mo *K*α radiation, λ = 0.71073 Å
Hall symbol: -P 2yn	Cell parameters from 25 reflections
*a* = 4.4760 (9) Å	θ = 10–13°
*b* = 13.654 (3) Å	µ = 0.52 mm^−^^1^
*c* = 12.400 (3) Å	*T* = 293 K
β = 97.16 (3)°	Block, colourless
*V* = 751.9 (3) Å^3^	0.30 × 0.20 × 0.10 mm
*Z* = 4	

### Data collection

**Table:**

Enraf–Nonius CAD-4 diffractometer	699 reflections with *I* > 2σ(*I*)
Radiation source: fine-focus sealed tube	*R*_int_ = 0.092
Graphite monochromator	θ_max_ = 25.4°, θ_min_ = 2.2°
ω/2θ scans	*h* = 0→5
Absorption correction: ψ scan (North *et al.*, 1968)	*k* = 0→16
*T*_min_ = 0.859, *T*_max_ = 0.950	*l* = −14→14
1578 measured reflections	3 standard reflections every 200 reflections
1394 independent reflections	intensity decay: 1%

### Refinement

**Table:**

Refinement on *F*^2^	Primary atom site location: structure-invariant direct methods
Least-squares matrix: full	Secondary atom site location: difference Fourier map
*R*[*F*^2^ > 2σ(*F*^2^)] = 0.052	Hydrogen site location: inferred from neighbouring sites
*wR*(*F*^2^) = 0.132	H-atom parameters constrained
*S* = 1.00	*w* = 1/[σ^2^(*F*_o_^2^) + (0.052*P*)^2^] where *P* = (*F*_o_^2^ + 2*F*_c_^2^)/3
1394 reflections	(Δ/σ)_max_ < 0.001
118 parameters	Δρ_max_ = 0.16 e Å^−^^3^
0 restraints	Δρ_min_ = −0.17 e Å^−^^3^

### Special details

**Table:**

Geometry. All e.s.d.'s (except the e.s.d. in the dihedral angle between two l.s. planes) are estimated using the full covariance matrix. The cell e.s.d.'s are taken into account individually in the estimation of e.s.d.'s in distances, angles and torsion angles; correlations between e.s.d.'s in cell parameters are only used when they are defined by crystal symmetry. An approximate (isotropic) treatment of cell e.s.d.'s is used for estimating e.s.d.'s involving l.s. planes.
Refinement. Refinement of *F*^2^ against ALL reflections. The weighted *R*-factor *wR* and goodness of fit *S* are based on *F*^2^, conventional *R*-factors *R* are based on *F*, with *F* set to zero for negative *F*^2^. The threshold expression of *F*^2^ > σ(*F*^2^) is used only for calculating *R*-factors(gt) *etc*. and is not relevant to the choice of reflections for refinement. *R*-factors based on *F*^2^ are statistically about twice as large as those based on *F*, and *R*- factors based on ALL data will be even larger.

### Fractional atomic coordinates and isotropic or equivalent isotropic displacement parameters (Å^2^)

**Table:**

	*x*	*y*	*z*	*U*_iso_*/*U*_eq_	
Cl	0.2118 (3)	0.00527 (9)	0.37810 (10)	0.1012 (5)	
F1	0.4799 (5)	0.18021 (17)	0.48010 (19)	0.0847 (7)	
C1	0.2598 (8)	0.2017 (3)	0.4004 (3)	0.0618 (10)	
O1	0.2232 (6)	0.4641 (2)	0.4113 (2)	0.0892 (9)	
O2	0.5635 (6)	0.3685 (2)	0.5008 (2)	0.0849 (9)	
H2A	0.6253	0.4209	0.5272	0.127*	
F2	−0.2511 (6)	0.0700 (2)	0.2054 (2)	0.1026 (9)	
C2	0.1144 (10)	0.1237 (3)	0.3435 (3)	0.0711 (11)	
C3	−0.1036 (10)	0.1438 (4)	0.2615 (3)	0.0768 (12)	
F3	−0.4110 (5)	0.2527 (2)	0.15189 (18)	0.0911 (8)	
C4	−0.1863 (9)	0.2388 (3)	0.2335 (3)	0.0687 (11)	
C5	−0.0428 (8)	0.3137 (3)	0.2896 (3)	0.0645 (10)	
H5A	−0.0972	0.3778	0.2707	0.077*	
C6	0.1844 (8)	0.2971 (3)	0.3749 (3)	0.0555 (9)	
C7	0.3271 (8)	0.3836 (3)	0.4317 (3)	0.0583 (9)	

### Atomic displacement parameters (Å^2^)

**Table:**

	*U*^11^	*U*^22^	*U*^33^	*U*^12^	*U*^13^	*U*^23^
Cl	0.1469 (12)	0.0629 (7)	0.0972 (9)	0.0035 (7)	0.0284 (7)	−0.0006 (7)
F1	0.1002 (18)	0.0701 (15)	0.0811 (14)	0.0138 (13)	0.0013 (13)	−0.0033 (13)
C1	0.068 (3)	0.067 (3)	0.051 (2)	0.006 (2)	0.0116 (19)	0.0000 (19)
O1	0.103 (2)	0.0606 (19)	0.095 (2)	0.0080 (16)	−0.0235 (17)	−0.0072 (16)
O2	0.096 (2)	0.0667 (18)	0.086 (2)	0.0034 (15)	−0.0111 (17)	−0.0161 (16)
F2	0.122 (2)	0.091 (2)	0.0952 (18)	−0.0351 (16)	0.0130 (15)	−0.0215 (16)
C2	0.092 (3)	0.061 (3)	0.066 (3)	−0.005 (2)	0.032 (2)	−0.004 (2)
C3	0.080 (3)	0.086 (3)	0.067 (3)	−0.026 (3)	0.020 (2)	−0.016 (3)
F3	0.0777 (15)	0.119 (2)	0.0715 (14)	−0.0059 (14)	−0.0103 (13)	−0.0029 (15)
C4	0.070 (3)	0.079 (3)	0.059 (2)	−0.011 (2)	0.014 (2)	−0.007 (2)
C5	0.068 (3)	0.070 (3)	0.057 (2)	−0.009 (2)	0.0156 (19)	−0.004 (2)
C6	0.056 (2)	0.060 (2)	0.053 (2)	−0.0027 (19)	0.0150 (17)	−0.0031 (18)
C7	0.069 (2)	0.055 (2)	0.050 (2)	0.004 (2)	0.0060 (18)	−0.0007 (17)

### Geometric parameters (Å, º)

**Table:**

Cl—C2	1.716 (4)	C2—C3	1.346 (6)
F1—C1	1.338 (4)	C3—C4	1.381 (6)
C1—C6	1.373 (5)	F3—C4	1.348 (4)
C1—C2	1.393 (5)	C4—C5	1.354 (5)
O1—C7	1.208 (4)	C5—C6	1.391 (5)
O2—C7	1.293 (4)	C5—H5A	0.9300
O2—H2A	0.8200	C6—C7	1.479 (5)
F2—C3	1.349 (4)		
			
F1—C1—C6	121.0 (3)	F3—C4—C3	118.2 (4)
F1—C1—C2	117.5 (4)	C5—C4—C3	119.1 (4)
C6—C1—C2	121.5 (3)	C4—C5—C6	121.4 (4)
C7—O2—H2A	109.5	C4—C5—H5A	119.3
C3—C2—C1	118.4 (4)	C6—C5—H5A	119.3
C3—C2—Cl	121.2 (4)	C1—C6—C5	117.8 (3)
C1—C2—Cl	120.4 (3)	C1—C6—C7	124.7 (3)
C2—C3—F2	120.0 (4)	C5—C6—C7	117.5 (3)
C2—C3—C4	121.8 (4)	O1—C7—O2	123.0 (4)
F2—C3—C4	118.2 (4)	O1—C7—C6	119.7 (3)
F3—C4—C5	122.7 (4)	O2—C7—C6	117.2 (4)
			
F1—C1—C2—C3	−178.7 (3)	F3—C4—C5—C6	178.7 (3)
C6—C1—C2—C3	0.3 (6)	C3—C4—C5—C6	−0.3 (5)
F1—C1—C2—Cl	1.5 (5)	F1—C1—C6—C5	178.8 (3)
C6—C1—C2—Cl	−179.5 (3)	C2—C1—C6—C5	−0.2 (5)
C1—C2—C3—F2	−179.4 (3)	F1—C1—C6—C7	−1.3 (5)
Cl—C2—C3—F2	0.4 (6)	C2—C1—C6—C7	179.7 (3)
C1—C2—C3—C4	−0.5 (6)	C4—C5—C6—C1	0.2 (5)
Cl—C2—C3—C4	179.3 (3)	C4—C5—C6—C7	−179.7 (3)
C2—C3—C4—F3	−178.6 (3)	C1—C6—C7—O1	−171.8 (4)
F2—C3—C4—F3	0.3 (6)	C5—C6—C7—O1	8.1 (5)
C2—C3—C4—C5	0.5 (6)	C1—C6—C7—O2	9.0 (5)
F2—C3—C4—C5	179.4 (3)	C5—C6—C7—O2	−171.1 (3)

### Hydrogen-bond geometry (Å, º)

**Table:**

*D*—H···*A*	*D*—H	H···*A*	*D*···*A*	*D*—H···*A*
O2—H2*A*···O1^i^	0.82	1.84	2.658 (4)	178

Symmetry code: (i) −*x*+1, −*y*+1, −*z*+1.
